# Vibronic Spectroscopy and Cationic Features of p-Diethynylbenzene: Insights into Electronic Conjugation and Accidental Resonances

**DOI:** 10.3390/molecules31101741

**Published:** 2026-05-20

**Authors:** Keke Zhang, Xiateng Qin, Yan Zhao, Rui Wang, Changyong Li, Suotang Jia

**Affiliations:** 1 State Key Laboratory of Quantum Optics Technologies and Devices, Institute of Laser Spectroscopy, Shanxi University, Taiyuan 030006, China; zhangkeke323@163.com (K.Z.); 18734558738@163.com (X.Q.); 19861241857@163.com (R.W.); tjia@sxu.edu.cn (S.J.); 2 Department of Physics and Electronics Engineering, Jinzhong University, Jinzhong 030619, China; zhaoy@jzxy.edu.cn; 3 Collaborative Innovation Center of Extreme Optics, Shanxi University, Taiyuan 030006, China

**Keywords:** p-diethynylbenzene, MATI spectroscopy, accidental resonance, vibrational analysis, REMPI, DFT calculations

## Abstract

We report a comprehensive spectroscopic investigation of p-diethynylbenzene (pDEB) using two-color resonance-enhanced multiphoton ionization (REMPI) and mass-analyzed threshold ionization (MATI) spectroscopy, complemented by density functional theory (DFT) calculations. The S_1_ ← S_0_ electronic origin is observed at 34,255 ± 2 cm^−1^. The adiabatic ionization energy (IE) is determined to be 69,095 ± 5 cm^−1^ from two-color MATI spectra recorded via the S_1_ origin. Notably, a narrow peak is observed at 32 cm^−1^ above the IE in the two-color MATI spectrum, which is assigned to a one-color, two-photon accidental resonance arising from the near-resonant condition where the S_1_ ← S_0_ transition energy (34,255 cm^−1^) is close to half of the IE (69,099/2 = 34,549.5 cm^−1^). This observation is consistent with similar reports for p-chlorofluorobenzene and p-difluorobenzene. Franck–Condon simulations show good agreement with the experimental spectra. The present results provide a spectroscopic basis for identifying pDEB and distinguishing it from its ortho and meta isomers.

## 1. Introduction

Diethynylbenzenes (DEBs) are important building blocks for the synthesis of novel conjugated polymers, molecular wires, and advanced optical materials [1,2,3]. Among the three isomers (ortho, meta, and para), p-diethynylbenzene (pDEB) has attracted particular interest due to its linear geometry and extended π-conjugation, which make it an ideal candidate for studying charge transport and electronic communication along molecular backbones [4,5]. Understanding the geometric and electronic structural changes in pDEB upon excitation and ionization is crucial for elucidating its charge transport mechanisms in molecular wire applications. Specifically, the adiabatic ionization energy (IE) and the vibrational frequencies of the cationic ground state (D_0_) serve as fundamental parameters for evaluating the stability and reactivity of charge carriers in pDEB-based materials. However, detailed spectroscopic characterization of pDEB in its electronically excited and cationic states remains limited, hindering a comprehensive understanding of its structure–property relationships.

The vibronic spectroscopy of pDEB has been previously investigated by Stearns and Zwier using resonant two-photon ionization (R2PI) and resonant ion-dip infrared spectroscopy [6]. They reported the S_1_ ← S_0_ origin at 34,255 cm^−1^ and identified several vibronic bands, with the spectrum dominated by false origins arising from vibronic coupling between the S_1_ (^1^B_2u_) and S_2_ (^1^B_1u_) states. However, a comprehensive vibrational analysis of the cation ground state (D_0_) has not been reported to date.

Resonance-enhanced multiphoton ionization (REMPI) [7,8,9,10,11] and mass-analyzed threshold ionization (MATI) spectroscopy [12,13,14,15,16] have proven to be powerful tools for investigating the vibronic structure of molecules in the gas phase. By combining supersonic jet cooling with mass-selective detection, these techniques allow unambiguous assignment of vibrational features for individual isomers, free from spectral congestion caused by impurities or conformational mixtures [17,18,19,20]. Moreover, MATI spectroscopy provides high-resolution information about the cation ground state (D_0_), including precise ionization energies (IEs) and vibrational frequencies [21,22,23,24,25,26,27,28].

An accidental resonance phenomenon is commonly observed in high-resolution MATI and ZEKE (zero kinetic energy) spectral analyses [29,30,31,32,33,34]. Such resonance signals typically appear as unexpected narrow peaks, whose width is significantly narrower than that of typical two-color MATI spectral bands, and cannot be assigned to a cation vibrational transition based on Franck–Condon considerations or calculated frequencies. Physically, this type of resonance originates from a one-color, two-photon excitation process, where the ionization laser alone (without the pump laser) excites the molecule from S_0_ to high-n Rydberg states via a single-color, two-photon process, followed by pulsed field ionization. Such accidental resonances are generally possible when the S_1_ ← S_0_ transition energy is close to half of the IE.

In this paper, we present a detailed spectroscopic investigation of pDEB using two-color R2PI and MATI techniques. A complete vibrational assignment is provided for both the S_1_ and D_0_ states. We discuss the observed accidental resonance in the MATI spectra and rationalize its occurrence based on the energy level structure of pDEB. Finally, we analyze the geometric changes upon electronic excitation and ionization with the aid of theoretical calculations.

## 2. Results

### 2.1. Two-Color R2PI Spectra of pDEB

**Figure 1:**
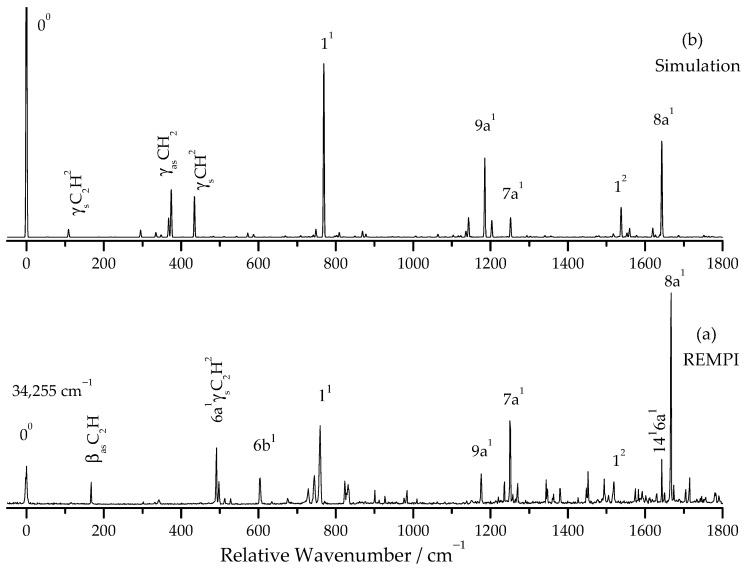
(**a**) Two-color R2PI spectrum of p-diethynylbenzene recorded in the vibrational frequency range of 0–1800 cm^−1^ (corresponding to excited energy: 34,000–36,100 cm^−1^) with the ionization laser fixed at 286 nm (34,965 cm^−1^). The S_1_ origin is observed at 34,255 cm^−1^. (**b**) The simulation spectra of the S_1_ ← S_0_0^0^ transition.

Figure 1a shows the two-color R2PI spectrum of pDEB in the vibrational frequency range of 0–1800 cm^−1^ (corresponding to excitation energies: 34,000–36,100 cm^−1^). The S_1_ ← S_0_ electronic origin is observed at 34,255 ± 2 cm^−1^, consistent with the value reported by Stearns and Zwier [6]. Table 1 presents the observed vibronic bands with their relative intensities, shifts from the origin, calculated frequencies, and assignments. The numbering system for ring vibrations follows Wilson notation [35,36], while substituent vibrations are designated using Greek letters with subscripts “s” and “as” to denote symmetric and antisymmetric motions, respectively.

The most intense bands in the spectrum are assigned to ring modes: 1^1^ (breathing) at 759 cm^−1^, 6b^1^ at 604 cm^−1^, 9a^1^ at 1176 cm^−1^, 7a^1^ at 1250 cm^−1^, and 8a^1^ at 1667 cm^−1^. These assignments are consistent with those reported for phenylacetylene [37] and p-diethynylbenzene [6]. Several low-frequency bands are observed below 500 cm^−1^, including features at 167, 302, 332, and 492 cm^−1^, which are assigned to out-of-plane and in-plane bending modes involving the ethynyl substituents.

### 2.2. Two-Color MATI Spectrum and Observation of an Accidental Resonance

**Figure 2:**
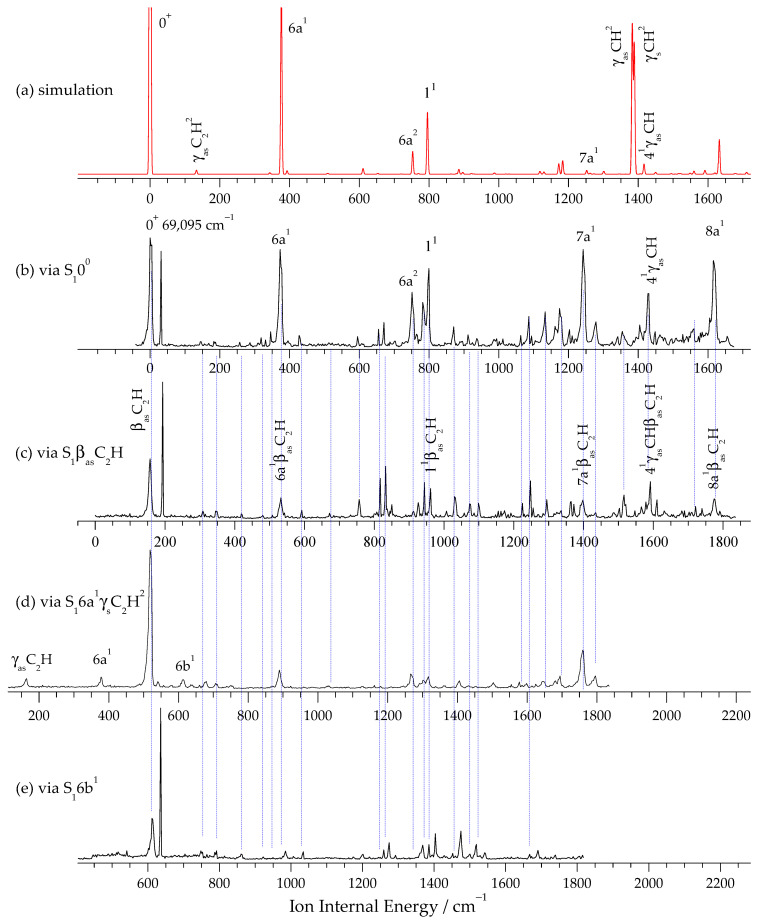
(**a**) Simulated D_0_ ← S_1_ 0^0^ spectrum of pDEB. (**b**–**e**) MATI spectra recorded via the intermediate states S_1_ 0^0^ (**b**), S_1_ β_as_C_2_H (**c**), S_1_ 6a^1^γ_s_C_2_H^2^ (**d**), and S_1_ 6b^1^ (**e**). The blue vertical lines indicate one-to-one correspondences. The MATI spectrum in (**d**) shows three additional fundamentals (γ_as_C_2_H, 6a, 6b), and the accidental resonance is absent in (**d**) due to competition from strong two-color MATI absorption.

Figure 2a shows the Franck–Condon simulation, which is in good agreement with the experimental MATI spectrum (Figure 2b), supporting the vibrational assignments. The simulated spectrum was generated using the TD-DFT-optimized geometries and frequencies for the S_1_ and D_0_ states, with appropriate scaling factors of 0.98.

Figure 2b shows the two-color MATI spectrum of pDEB recorded by ionizing through the S_1_0^0^ level (34,255 cm^−1^). The spectrum exhibits a strong origin band, which corresponds to an adiabatic ionization energy (IE) of 69,095 ± 5 cm^−1^ (including a Stark shift correction of +4√F = +4√0.9 ≈ +3.8 cm^−1^). This IE value is in excellent agreement with the G4 and CBS-QB3 predictions (69,138 and 69,141 cm^−1^, respectively).

The vibrational frequencies of the pDEB cation (D_0_ state) are summarized in Table 2. The most intense bands correspond to totally symmetric ring modes, including 1^1^ at 799 cm^−1^, 6a^1^ at 373 cm^−1^, and 9a^1^ at 1203 cm^−1^. The frequencies of these modes in the D_0_ state are slightly higher than those in the S_1_ state, reflecting the increased force constants upon removal of a π electron. For example, the breathing mode (1^1^) shifts from 759 cm^−1^ in S_1_ to 799 cm^−1^ in D_0_.

In Figure 2b, an unexpected narrow peak is observed at approximately 32 cm^−1^ above the cationic origin 0^+^. This peak has a full width at half-maximum (FWHM) of approximately 2–3 cm^−1^, which is significantly narrower than the typical two-color MATI bands (FWHM ~7–10 cm^−1^) and cannot be assigned to any cation vibrational transition (D_0_ ← S_1_ 0^0^) based on Franck–Condon calculations or known vibrational frequencies of the pDEB cation. This phenomenon has been observed and analyzed in detail for several molecules. The underlying principle involves the ground-state molecule simultaneously absorbing two photons of the same frequency from the ionization laser (or a single strong laser) to reach Rydberg states, followed by pulsed-field ionization and detection. That is, it arises from a one-color, two-photon process. The probability of two-photon absorption is significant only under near-resonant conditions, making it readily observable. Specifically, this occurs when a molecule possesses an actual energy level very close to half the energy difference between the Rydberg state and the electronic ground state. Timothy G. Wright’s group has conducted detailed studies on this phenomenon, referring to it as an accidental resonance [31].

Figure 2c shows the MATI spectrum recorded via the intermediate state S_1_ β_as_C_2_H. This MATI spectrum is assigned to combination vibrations involving the β_as_C_2_H intermediate-state mode and the MATI-active modes observed via S_1_ 0^0^. The one-to-one correspondence between them is indicated by blue vertical lines in the figure. This implies that the ion retains the motion of the intermediate state. The accidental resonance is also observed. The strongest MATI peak appears at a shift of 158 cm^−1^ and is assigned to the cationic in-plane bending mode of the ethynyl group, β_as_C_2_H, consistent with the intermediate state mode. This Δν = 0 propensity rule indicates that the molecular structure does not undergo significant change upon transition from the excited state to the cationic ground state.

Figure 2d presents the MATI spectrum measured via the relatively strong intermediate state S_1_ 6a^1^γ_s_C_2_H^2^, achieving excellent signal-to-noise ratio and resolution. Similar to Figure 2c, apart from three new fundamental vibrations (γ_as_C_2_H, 6a, 6b) observed on the low-frequency side, all other vibrational bands are assigned to combination vibrations involving the intermediate-state mode 6a^1^γ_s_C_2_H^2^ and the MATI-active modes observed via S_1_ 0^0^. Another notable difference is that the MATI signal intensity is more than double that obtained via other intermediate states, while the accidental resonance line is not observed. This is likely due to a competitive effect between accidental resonance and conventional MATI. As can be seen from the REMPI spectrum in Figure 1a, the strong signal of this intermediate state indicates a large transition dipole moment from the ground state to this vibrational mode, resulting in a high population. Consequently, more molecules absorb the ionizing photon to reach the Rydberg state, leading to a stronger MATI signal. In other words, the ionizing photon is heavily absorbed during the two-color MATI process, making the one-color two-photon absorption—a nonlinear process required for accidental resonance—nearly impossible due to insufficient light intensity. Therefore, the accidental resonance line is not observed.

Figure 2e, similar to Figure 2c, shows no new fundamental vibrations; all signals can be assigned to combination vibrations of the intermediate-state mode 6b with the vibrations observed in Figure 2b.

## 3. Discussion

### 3.1. Molecular Geometry in the S_0_, S_1_, and D_0_ States

**Figure 3:**
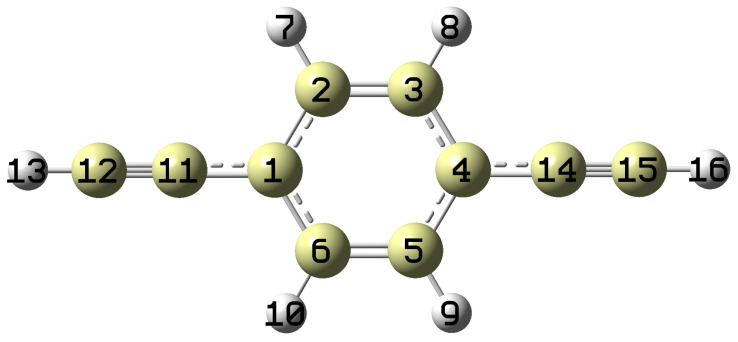
Atom labeling for p-diethynylbenzene used in the geometric parameter calculations (Table 3). The carbon atoms of the benzene ring are numbered C1–C6, the ethynyl carbon atoms are C11, C12, C14, C15, and the hydrogen atoms are H13 and H16.

The optimized geometries of pDEB in the S_0_, S_1_, and D_0_ states are summarized in Table 3, with the atom labeling shown in Figure 3. Upon S_1_ ← S_0_ excitation, the C1–C2, C3–C4, C4–C5, and C6–C1 bonds (the long axes of the ring) elongate by 0.04 Å, while the C2–C3 and C5–C6 bonds (the short axes) contract by 0.02 Å. This pattern indicates a quinoidal distortion of the ring upon π → π* excitation, which is characteristic of para-disubstituted benzenes with electron-withdrawing or conjugated substituents [6]. The C1–C11 and C4–C14 bonds (connecting the ring to the ethynyl groups) shorten by 0.035 Å, indicating increased conjugation between the ring and the substituents in the excited state. The C≡C triple bonds elongate slightly by 0.021 Å, while the C–H bonds remain essentially unchanged.

Upon ionization (D_0_ ← S_1_), most geometric parameters revert toward their S_0_ values. The ring bonds that elongated in S_1_ contract slightly (by 0.017 Å), while the C1–C11 and C4–C14 bonds elongate slightly (by 0.004 Å). The C≡C bonds contract by 0.011 Å, approaching their S_0_ lengths. The bond angles show complementary changes: the C3–C4–C5 and C6–C1–C2 angles decrease by 0.44° in S_1_ and increase by 1.52° in D_0_, reflecting the rehybridization of the ring carbons upon electronic excitation and ionization.

These geometry changes provide direct evidence of the electronic redistribution upon excitation and ionization. The observed quinoidal distortion and the shortening of the C(ring)-C(ethynyl) bonds in the S_1_ state indicate a significant enhancement of π-conjugation between the benzene ring and the ethynyl substituents. This structural rigidity and extended conjugation are key features that facilitate efficient charge transport in pDEB-based molecular wires. Furthermore, the reversion of bond lengths in the D_0_ state suggests a different electronic distribution mechanism upon electron removal, which is critical for understanding the hole-transport properties of this molecule.

### 3.2. Vibrational Analysis of the S_1_ and D_0_ States

pDEB (C_10_H_6_) contains 16 atoms and has 42 normal vibrational modes. Following the approach of Varsányi [35,36], we classify these modes into two categories: (1) ring vibrations (30 modes) that resemble those of benzene, labeled using Wilson notation [38]; and (2) substituent vibrations (12 modes) associated with the two –C≡CH (or C_2_H)groups.

For the substituent vibrations, each –C≡CH group has six normal modes: ν(C–C≡C) stretching, β(C≡C–H) in-plane bending, γ(C≡C–H) out-of-plane bending, ν(C–H) stretching, β(C–H) in-plane bending, and γ(C–H) out-of-plane bending. Since pDEB has two equivalent ethynyl groups in para positions, each of these six vibrations splits into symmetric (s) and antisymmetric (as) components. We designate these using Greek letters with subscripts “s” and “as”: ν_s_, ν_as_; β_s_, β_as_; γ_s_, γ_as_; etc.

The assignment of substituent modes is based on comparisons with phenylacetylene [37] and diethynylbenzene [6], as well as on TD-DFT frequency calculations. For example, the band at 167 cm^−1^ is assigned to β_as_C_2_H (antisymmetric in-plane bending of the ethynyl, while the band at 492 cm^−1^ is assigned to 6a^1^γ_as_C_2_H^2^ (a combination of ring mode 6a with two quanta of antisymmetric out-of-plane C≡C–H bending). The band at 1176 cm^−1^ is assigned to 9a^1^ (ring mode 9a, in-plane C–H bending), which is consistent with the assignment for p-diethynylbenzene reported by Stearns and Zwier [6].

For the D_0_ state cation, the vibrational frequencies are generally higher than those in S_1_, indicating stronger bonding upon ionization. For instance, the ring breathing mode (1^1^) increases from 759 cm^−1^ in S_1_ to 799 cm^−1^ in D_0_, and the 6b^1^ mode increases from 605 cm^−1^ to 613 cm^−1^. This trend is consistent with the removal of an electron from a π orbital, which increases the effective nuclear charge experienced by the remaining electrons and strengthens the σ framework.

### 3.3. Accidental Resonances

The observation of accidental resonances in two-color MATI spectra has been reported for several molecules where the S_1_ ← S_0_ transition energy is close to half of the IE. This phenomenon occurs for pDEB because the S_1_ ← S_0_ transition energy of pDEB (34,255 cm^−1^) is very close to half of the IE (69,099/2 = 34,549.5 cm^−1^). Similar observations have been reported for ethyl bromide by Tang et al. [32] for ethyl iodide by Knoblauch et al. [33], and for p-difluorobenzene and p-chlorofluorobenzene by Kemp et al. [30,31]. In the latter study, Wright and co-workers observed “one-colour, two-photon accidental resonances” in ZEKE spectra of p-difluorobenzene and p-chlorofluorobenzene and assigned them to similar resonant conditions. Table 4 summarizes the relevant energy parameters for pDEB and related molecules.

For p-difluorobenzene (pDFB), Kemp et al. [30] reported similar accidental resonances in ZEKE spectra and attributed them to “one-colour, two-photon accidental resonances” where the ionization laser becomes resonant with an S_1_ ← S_0_ transition and additionally, the fixed excitation laser is then accidentally resonant with a transition from this S_1_ level to a level in the cation. The near-resonant condition in pDFB (IE/2 − S_1_ = 97 cm^−1^) is even closer than in pDEB (295 cm^−1^), leading to intense accidental signals.

For ethyl bromide, Tang et al. [32] systematically investigated one-color, two-photon MATI spectroscopy and observed extensive vibrational structure arising from resonance enhancement by a dissociative intermediate state (the Ã state). The A-band of ethyl bromide centers at approximately 200 nm (50,000 cm^−1^), which is roughly half of the IE (83,099 cm^−1^), making the molecule particularly suitable for one-color, two-photon studies.

The narrow linewidth of the accidental resonance (~2–3 cm^−1^) compared to the two-color MATI bands (~7–10 cm^−1^) can be understood by considering the different ionization mechanisms. Two-color MATI involves excitation to a specific intermediate level (S_1_ 0^0^) followed by a second photon to high-n Rydberg states, with rotational congestion contributing to broader linewidths. In contrast, the one-color, two-photon accidental resonance arises from a direct two-photon absorption transition from S_0_ to high-n Rydberg states via the near-resonant S_1_ level, which can exhibit narrower linewidths due to more stringent selection rules.

It is worth noting that similar one-color, two-photon accidental resonances have also been observed in alkyl halides such as ethyl bromide [32], 1-bromopropane [34] and 2-bromopropane [29], where dissociative intermediate states provide the near-resonant condition. This suggests that accidental resonances may be a general phenomenon in MATI spectroscopy when the energy of an accessible intermediate state (either bound or dissociative) lies close to half the ionization energy.

**Figure 4:**
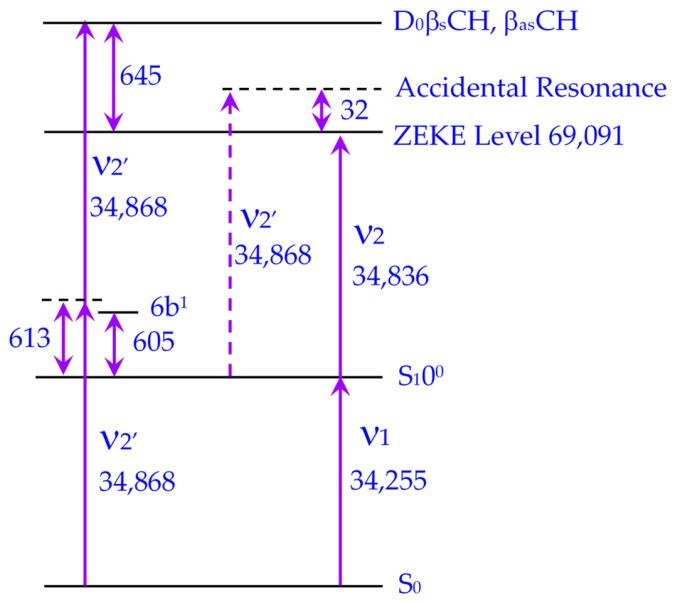
Energy level diagram (unit: cm^−1^) illustrating the origin of the one-color, two-photon accidental resonance in pDEB.

These comparative studies support our assignment of the narrow peak at 32 cm^−1^ above the IE in the pDEB MATI spectrum as a one-color, two-photon accidental resonance. The phenomenon arises from the specific energy level structure of pDEB (see Figure 4), where the S_1_ states 6b^1^ lie approximately halfway between S_0_ and D_0_β_s_CH, β_as_CH (The calculated frequencies of both are 650 cm^−1^), making the molecule susceptible to such resonances.

## 4. Materials and Methods

### 4.1. Sample Preparation and Molecular Beam

The pDEB sample (97% purity, purchased from Macklin Biochemical Co., Ltd., Shanghai, China) was placed in a sample reservoir heated to approximately 108 °C to achieve sufficient vapor pressure. The sample vapor was seeded in 3 bar of krypton (Kr) carrier gas and expanded through a pulsed valve (0.5 mm diameter orifice) into the source chamber. The resulting molecular beam was skimmed (1.0 mm diameter) before entering the ionization chamber. The pressures in the source and ionization chambers were maintained at approximately 1 × 10^−4^ Pa and 1 × 10^−5^ Pa, respectively.

### 4.2. Laser Systems and Spectroscopy

Two tunable UV laser systems were used for two-color R2PI experiments. The probe laser (excitation) was a Nd:YAG-pumped dye laser (CBR-D-24, Sirah Lasertechnik GmbH, Gottingen, Lower Saxony, Germany) with frequency-doubled output (277.5–293 nm). The ionization laser was a Nd:YAG-pumped dye laser (Precision Scan-D, Sirah Lasertechnik GmbH, Gottingen, Lower Saxony, Germany) with frequency-doubled output (fixed at 286 nm for two-color REMPI). Laser wavelengths were calibrated using a wavemeter (High-FinesseWS-7, HighFinesse GmbH, Offenburg, Germany).

Ions were accelerated by a two-stage electric field and detected by a microchannel plate (MCP) detector after passing through a 48 cm field-free region. Ion signals were processed by a multichannel scaler (SR430, Stanford Research Systems, Inc., Sunnyvale, CA, USA) and recorded by a personal computer using a self-written LabVIEW program. Mass spectra were accumulated at 0.02 nm intervals for R2PI and 0.04 nm intervals for MATI, with 300 laser shots per data point.

For MATI experiments, a pulsed electric field of −0.9 V/cm was applied approximately 180 ns after the laser pulses to reject prompt ions. After a delay of ~11.8 μs, a second pulsed field of +143 V/cm was applied to field-ionize long-lived high-n Rydberg states. The resulting threshold ions were detected in the same manner as prompt ions. Further experimental details can be found in our previously published papers [40,41].

### 4.3. Computational Methods

All calculations were performed using the Gaussian 16 software package [42]. Ground state (S_0_) geometries and harmonic vibrational frequencies were calculated using density functional theory (DFT) at the B3LYP/6-31G(d) level. Excited state (S_1_) geometries and frequencies were calculated using time-dependent DFT (TD-DFT) at the same level of theory. Cation ground state (D_0_) calculations were performed using unrestricted DFT (UB3LYP/6-31G(d)). The average value of S^2^ in the D_0_ state is 0.75 after spin annihilation (ca. 0.77 before annihilation), confirming that spin contamination is negligible and the use of UB3LYP is well justified. All calculated frequencies were scaled by appropriate factors (0.991 for S_1_, 0.98 for D_0_) to correct for basis set incompleteness and anharmonicity. The scaling factor is empirical and chosen to achieve the best consistency between calculated frequencies and experimental measurements. Based on the DFT results for the ground, excited, and cationic ground states, we performed simulations of the REMPI and MATI spectra using Gaussian 16. The spectral linewidths were set according to the experimental values (REMPI: SpecHwHm = 1.4 cm^−1^; MATI: FWHM = 2.3 cm^−1^). The spectral resolution (step size) was 0.2 cm^−1^. The transition from the ground state to the excited state included the first-order correction of the Taylor expansion, i.e., the Herzberg–Teller correction. The adiabatic excitation energy was determined from the calculated energies of the two relevant electronic states.

To provide accurate predictions that can guide experimental work, G4 and CBS-QB3 methods were employed. The calculated ionization energies are essential for determining the optimal laser wavelengths and selecting appropriate dyes, which significantly enhances experimental efficiency.

## 5. Conclusions

We have performed a detailed spectroscopic investigation of p-diethynylbenzene using two-color R2PI and MATI techniques. The S_1_ ← S_0_ electronic origin is located at 34,255 ± 2 cm^−1^, and the adiabatic ionization energy is determined to be 69,095 ± 5 cm^−1^ from two-color MATI spectroscopy via the S_1_ 0^0^ level. A complete vibrational analysis of the S_1_ and D_0_ states is presented. The assignments are supported by TD-DFT frequency calculations and Franck–Condon simulations.

An unexpected narrow peak at 32 cm^−1^ above the ionization threshold is observed in the two-color MATI spectrum and is assigned to a one-color, two-photon accidental resonance. This is consistent with similar reports for ethyl bromide [32], ethyl iodide [33], p-difluorobenzene [30] and p-chlorofluorobenzene [31], suggesting that accidental resonances are a general phenomenon in MATI/ZEKE spectroscopy of molecules with S_1_ states lying approximately halfway between S_0_ and D_0_.

The present results provide a comprehensive spectroscopic dataset and precise structural parameters for pDEB. These data not only allow for the unambiguous identification of pDEB and its distinction from ortho and meta isomers but also serve as a benchmark for theoretical modeling of conjugated molecular wires and advanced optical materials.

## Figures and Tables

**Table 1:** Vibrational frequencies (in cm^−1^) and tentative assignments of observed bands in the REMPI experiment of p-Diethynylbenzene ^a^.

Energy	Shift	Rel. Int.	Cal.	Assignment ^b^	Energy	Shift	Rel. Int.	Cal.	Assignment ^b^
34,255	0	18	0	S_1_ Origin, 0^0^	35,491	1236	11	1233	γ_s_CH^4^γ_as_C_2_H^2^
34,422	167	11	169	β_as_C_2_H	35,505	1250	39	1252	7a^1^
34,557	302	1	296	γ_as_C_2_H^2^	35,513	1258	5	1259	8b^1^γ_s_C_2_H^2^
34,587	332	1	333	γ_as_C_2_Hγ_as_CH	35,526	1271	10	1269	17b^1^16b^1^
34,598	343	2	348	11^1^γ_s_C_2_H	35,599	1344	12	1342	1^1^10b^1^γ_as_C_2_H
34,747	492	27	477	6a^1^γ_s_C_2_H^2^	35,618	1363	5	1361	7a^1^γ_s_C_2_H^2^
34,753	498	11	480	γ_as_CH^2^γ_s_C_2_H^2^	35,634	1379	8	1378	16b^2^γ_as_CH^2^
34,768	513	3	509	11^1^γ_s_CH	35,681	1426	3	1426	17b^1^6a^1^11^1^
34,783	528	3	540	γ_s_CH^2^γ_s_C_2_H^2^	35,707	1452	15	1449	11^2^γ_s_CH^4^
34,860	605	12	615	6b^1^	35,749	1494	12	1495	1^1^γ_s_CH^2^γ_as_CH^1^
34,890	635	1	629	γ_as_CHγ_as_C_2_H^3^	35,761	1506	4	1510	1^1^γ_as_CH^4^
34,930	675	3	675	4^1^	35,773	1518	11	1518	1^2^
34,984	729	7	727	γ_s_CH^2^γ_as_C_2_H^2^	35,830	1575	7	1571	1^1^γ_s_CH^2^γ_as_CH^2^
35,000	745	14	742	γ_as_CH^4^	35,837	1582	7	1585	7a^1^γ_as_CH^1^γ_as_C_2_H
35,014	759	37	769	1^1^	35,848	1593	6	1590	1^1^17b^1^γ_s_C_2_H
35,027	772	2	780	11^1^γ_s_CH^2^γ_s_C_2_H	35,885	1630	5	1616	9a^1^γ_s_CH^2^
35,078	823	11	820	17b^1^γ_s_C_2_H	35,898	1643	21	1623	7a^1^γ_as_CH^2^
35,087	832	9	829	11^1^γ_as_CH^2^γ_s_C_2_H^3^	35,906	1651	5	1631	1^1^γ_s_CH^4^
35,156	901	7	906	16b^1^1^11^γ_s_C_2_H^2^	35,922	1667	100	1643	8a^1^
35,167	912	2	911	10b^1^β_as_C_2_H^2^γ_as_C_2_H	35,929	1674	9	1668	8b^1^9b^1^
35,182	927	4	930	17b^1^γ_s_C_2_H^3^	35,960	1705	7	1699	1^1^17b^1^γ_s_C_2_H^3^
35,231	976	3	972	γ_s_CH^4^γ_s_C_2_H^2^	35,970	1715	13	1712	10b^2^γ_s_CH^4^
35,238	983	7	987	1^1^γ_s_CH^4^	36,001	1746	4	1752	8a^1^γ_s_C_2_H^2^
35,265	1010	3	1007	16b^2^	36,012	1757	3	1758	9a^2^10b^1^γ_as_C_2_H
35,431	1176	14	1185	9a^1^	36,038	1780	5	1772	9a^1^11^2^
35,475	1220	3	1220	10b^2^γ_as_C_2_H^2^	36,045	1790	4	1788	1^1^11^2^γ_s_CH^2^

^a^ The experimental frequencies are shifts from 34,255 cm^−1^, whereas the predicted values are obtained from the td-B3LYP/6-31G(d) calculations, scaled by 0.991. ^b^ Internal vibrations of the substituents: β, in-plane bending; γ, out-of-plane bending; the subscript ‘s’ and ‘as’, symmetric and antisymmetric motion, respectively.

**Table 2:** Vibrational frequencies (in cm^−1^) and tentative assignments of observed bands in the MATI experiment of PDEB ^a^.

Intermediate State				Assignment ^b^	Intermediate State				Assignment ^b^
0^0^	β_as_C_2_H	6a^1^γ_s_C_2_H^2^	Calc.		0^0^	β_as_C_2_H	6a^1^ γ_s_C_2_H^2^	Calc.	
0			0	D_0_ Origin, 0^+^	871			878	10b^2^
32				accidental resonance	912			921	17b^1^γ_s_C_2_H
146			133	γ_s_C_2_H^2^	937			928	1^1^γ_s_C_2_H^2^
	158		173	β_as_C_2_H	986			987	10b^1^6a^1^γ_as_C_2_H
		165	172	γasC_2_H	1012			1021	11^1^γ_s_CH
257			234	β_s_C_2_H^2^	1063			1063	9b^2^
286			266	γ_s_C_2_H^4^	1086			1081	9b^1^6a^1^ β_as_C_2_H
318			316	γ_s_C_2_H^3^β_s_C_2_H	1133			1118	16b^2^
331			327	11^1^				1130	6a^3^
346			343	γ_as_C_2_H^2^		960			1^1^β_as_C_2_H
374		377	377	6a^1^	1161			1164	4^1^10b^1^
395			393	11^1^γ_s_C_2_H	1174			1172	1^1^6a^1^
427				γ_s_C_2_H^6^	1203			1183	9a^1^
	532			6a^1^β_as_C_2_H	1242			1252	7a^1^
595			610	10b^1^γ_as_C_2_H	1278			1273	4^1^6a^1^γ_as_C_2_H
		613	618	6b^1^	1354			1346	17a^1^16a^1^
655			654	11^2^		1398			7a^1^β_as_C_2_H
670				11^1^γ_as_C_2_H^2^	1430			1416	4^1^γ_as_CH
702			705	9b^1^β_as_C_2_H		1592			4^1^γ_as_CHβ_as_C_2_H
751			753	6a^2^	1615			1632	8a^1^
782			786	11^2^γ_s_C_2_H^2^	1655			1673	1^1^10b^2^
799			796	1^1^		1774			8a^1^β_as_C_2_H

^a^ Experimental values are shifts from 69,095 cm^−1^. Calculated values are from UB3LYP/6-31G(d) scaled by 0.98. ^b^ Internal vibrations of the substituents: β, in-plane bending; γ, out-of-plane bending; the subscript ‘s’ and ‘as’, symmetric and antisymmetric motion. The 32 cm^−1^ peak is assigned to a one-color, two-photon accidental resonance.

**Table 3:** Optimized geometric parameters of pDEB in the S_0_, S_1_, and D_0_ states (atom labels as in Figure 3) calculated at the B3LYP/6-31G(d), TD-B3LYP/6-31G(d), and UB3LYP/6-31G(d) levels of theory, respectively.

	S_0_	S_1_	D_0_	Δ(S_1_ − S_0_)	Δ(D_0_ − S_1_)
Bond length (Å)					
C1–C2	1.409	1.449	1.432	0.04	−0.017
C2–C3	1.388	1.368	1.371	−0.02	0.003
C3–C4	1.409	1.449	1.432	0.04	−0.017
C4–C5	1.409	1.449	1.432	0.04	−0.017
C5–C6	1.388	1.368	1.371	−0.02	0.003
C6–C1	1.409	1.449	1.432	0.04	−0.017
C1–C11	1.428	1.393	1.397	−0.035	0.004
C4–C14	1.428	1.393	1.397	−0.035	0.004
C11≡C12	1.21	1.231	1.22	0.021	−0.011
C14≡C15	1.21	1.231	1.22	0.021	−0.011
C12–H13	1.066	1.067	1.071	1 × 10^−3^	0.004
C15–H16	1.066	1.067	1.071	1 × 10^−3^	0.004
Bond angle (°)					
C1–C2–C3	120.60	120.82	120.06	+0.22	−0.76
C2–C3–C4	120.60	120.82	120.06	+0.22	−0.76
C3–C4–C5	118.80	118.36	119.88	−0.44	+1.52
C4–C5–C6	120.60	120.82	120.06	+0.22	−0.76
C5–C6–C1	120.60	120.82	120.06	+0.22	−0.76
C6–C1–C2	118.80	118.36	119.88	−0.44	+1.52
C2–C1–C11	120.60	120.82	120.06	+0.22	−0.76
C6–C1–C11	120.60	120.82	120.06	+0.22	−0.76
C3–C4–C14	120.60	120.82	120.06	+0.22	−0.76
C5–C4–C14	120.60	120.82	120.06	+0.22	−0.76
C1–C11≡C12	180.00	180.00	180.00	0.00	0.00
C4–C14≡C15	180.00	180.00	180.00	0.00	0.00

**Table 4:** Comparison of S_1_ energies and IEs for molecules exhibiting one-color, two-photon accidental resonances (cm^−1^).

Molecule	S_1_	IE	IE/2	Δ(IE/2 − S_1_)	Reference
pDEB	34,255	69,099	34,550	+295	This work
pDFB	36,838	73,869	36,935	+97	[30]
p-Chlorofluorobenzene	36,275	72,919	36,644	+185	[31]
Ethyl bromide	~38,800	83,099	41,550	+2750	[32,39]
Ethyl iodide	~38,800	75,406	37,703	−1097	[33]

## Data Availability

The data that support the findings of this study are available from the corresponding author, Changyong Li, upon reasonable request.
